# An investigation into the knowledge, perceptions and role of personal protective technologies in Zika prevention in Colombia

**DOI:** 10.1371/journal.pntd.0007970

**Published:** 2020-01-21

**Authors:** Carolina Mendoza, Gloria-Isabel Jaramillo, Thomas H. Ant, Grace M. Power, Robert T. Jones, Juliana Quintero, Neal Alexander, Jayne Webster, Lyda Osorio, James G. Logan

**Affiliations:** 1 Epidemiology and Population Health Research Group, School of Public Health, Universidad del Valle, Universidad del Valle Campus San Fernando, Cali, Colombia; 2 School of Medicine, Universidad Cooperativa de Colombia, Villavicencio, Colombia; 3 Department of Disease Control, Faculty of Infectious and Tropical Diseases, London School of Hygiene and Tropical Medicine, London, United Kingdom; 4 Arthropod Control Product Test Centre (ARCTEC), Chariot Innovations Ltd, London School of Hygiene and Tropical Medicine, London, United Kingdom; 5 Department of Infectious Disease Epidemiology, Faculty of Epidemiology and Population Health, London, United Kingdom; Fundaçao Oswaldo Cruz, BRAZIL

## Abstract

**Background:**

Arboviruses transmitted by day-biting *Aedes* mosquitoes are a major public health concern. With the challenges inherent in arbovirus vaccine and therapeutics development, vector control and bite prevention strategies are among the limited options available for immediate intervention. Bite prevention through personal protective technologies (PPT), such as topical mosquito repellents or repellent-impregnated clothing, may help to decrease biting rates and, therefore, the risk of disease in groups most susceptible to adverse outcomes from Zika virus. However, achieving high uptake and compliance with PPT can be challenging.

**Methodology/Principal findings:**

To gain an insight into the knowledge and concerns of pregnant women surrounding Zika and their opinions regarding PPT, particularly repellent clothing, a focus group study was carried out with pregnant women, women of reproductive age, and semi-structured interviews with their male partners in two cities in Colombia. The discussions revealed shortfalls in basic knowledge of Zika virus, with several pregnant participants reporting being unaware of the potential for Zika-related congenital malformations. Although participants generally considered Zika to be a significant personal threat, most rated it as less of a concern than dengue or diarrheal diseases. Overall, repellent clothing and other forms of PPT were viewed as effective, although some participants expressed concerns over the high costs of repellents, and safety fears of regular contact with repellent chemicals, which they perceived as potentially harmful. Plant-derived repellents were considered to be safer than synthetic chemical repellents. Discussions also highlighted that health centers were the preferred source of information on bite-reduction.

**Conclusions/Significance:**

Achieving high uptake and compliance with PPT in populations most at risk of adverse outcomes from Zika infection requires engaging key users in open dialogue to identify and address any practical issues regarding PPT use, and concerns over safety. The findings presented here suggest that educational campaigns should strongly emphasize the risks associated with Zika during pregnancy, and discuss safety profiles of approved synthetic repellents and the availability of EPA-approved plant-based repellents. In addition, the economic and political context should be a major consideration when evaluating personal mosquito-repellent strategies.

## Author summary

Although mosquito personal protective technologies (PPT), such as topical DEET or permethrin impregnated clothing, show high efficacy in laboratory studies, they frequently achieve poor population uptake. This is likely due to numerous factors, including high costs, dislike of the feel or smell of the product, and safety fears over the repellent chemicals. The targeted use of PPT to those at elevated risk of severe disease, such as pregnant women in Zika endemic regions, may help mitigate adverse outcomes from Zika virus infection. To explore ways of increasing the appeal of PPT, a focus group study was performed with pregnant women and women of reproductive age in two cities in Colombia. Although almost all participants had heard of Zika, not all pregnant women were aware of the potential for Zika-related birth defects. PPT products were generally viewed as effective, although many expressed fears that repeated exposure to chemicals may affect fetal development. There was limited trust over repellent-manufacturer claims of safety, and women from low socioeconomic groups believed PPT products to be unaffordable. Participants identified health centers as trusted sources of bite-prevention education, suggesting health workers should emphasize safety and efficacy of approved repellents.

## Introduction

An epidemic outbreak of Zika virus was declared in Colombia in early October 2015 [1]. Approximately 108,000 suspected clinical cases were reported between August 2015 and November 2016, which included nearly 20,000 cases in pregnant women (~18%) [1]. Cali and Villavicencio experienced incidences above the national average, with 633.8 and 444.2 per 100,000 inhabitants, respectively. In Cali, the third largest city in Colombia with approximately 2.5 million inhabitants, a total of 15,181 Zika cases were reported [2]. In Villavicencio, a city with just over 500,000 inhabitants, 2278 Zika cases were reported to the public health surveillance system [3].

In the post-epidemic year (2017), 2130 cases were reported, of which 368 (17.2%) were in pregnant women [4]. While the Zika virus usually causes a mild and self-limiting febrile illness, the recent outbreak revealed a concomitant rise in fetal birth defects for women infected during pregnancy. The prevalence of congenital microcephaly in Colombia quadrupled during the epidemic to 11.6 cases per 10,000 births[5]. Zika infection has also been associated with a variety of neurological and autoimmune complications in adults, including encephalitis, Guillain-Barre Syndrome[6, 7] and immune-mediated thrombocytopenia[8].

The Zika virus is a flavivirus, closely related to dengue, and is principally transmitted by the bite of infected mosquitoes of the *Aedes* genus. Sexual contact is a secondary transmission route [9]. *Aedes aegypti* and *Aedes albopictus* are competent Zika vectors [10] and are abundant in Colombia [11–13], with the former species responsible for most cases of urban Zika transmission [14]. *Aedes aegypti* is a highly anthropophilic and aggressive day-time biter, often taking multiple human blood-meals in a short space of time [15]. Control of these vectors in Colombia largely focuses on the larviciding of domestic water containers and the cleaning-up of refuse that can collect water and act as larval breeding sites [16], with the addition of emergency-response insecticidal spraying when outbreaks of vector-borne diseases are detected [17, 18].

With a lack of treatment for Zika and its associated pathologies, measures that can effectively reduce transmission are critical. Such measures include government-led or individual/community-based mosquito population management, and the use of personal protective technologies (PPT). A key draw to PPT is its ease of use by individuals, enabling a sense of control over mosquito bite prevention. During the recent Zika epidemic, the Colombian Ministry of Health and Social Protection recommended that women avoid or postpone pregnancy [19]. This advice was viewed as insufficient and the government subsequently issued further guidance for pregnant women that included the promotion of measures that reduce vector contact, such as the wearing of long sleeves and trousers, and the topical application of recommended repellents, containing DEET, Picaridin or IR3535, among others [19]. The use of certain U.S. Environmental Protection Agency (EPA)-registered topical repellents for Zika prevention was also strongly recommended by the U.S. Centers for Disease Control and Prevention (CDC), together with the wearing of clothing impregnated with the insecticide permethrin [20]. However, achieving high uptake and compliance with PPT has been challenging historically [21], indicating a need to engage target users in open dialogue to identify and address any practical issues regarding PPT use, and concerns over safety. This is particularly apparent within groups of pregnant women, who have been observed to be wary of contact with chemicals perceived as potentially harmful [22].

A qualitative research study was conducted involving pregnant or reproductive-age women and their partners in the Colombian cities of Villavicencio and Cali to: 1) gain an understanding of the perceived importance of Zika and other arboviruses in this setting, 2) identify context specific knowledge of Zika transmission and pathology, 3) discern routine and desired mosquito abatement activities, and 4) explore perceptions and concerns regarding PPT—particularly mosquito-repellent/insecticide impregnated clothing.

## Methods

### Study design and population

A qualitative study was conducted in the Colombian cities of Cali (situated in the south-west) and Villavicencio (situated in central-east) in 2018. Both locations have a tropical climate, with average daily mean temperatures ranging from 23.4–24.2°C and 24.2–26.7°C throughout the year in Cali and Villavicencio, respectively (meteorological data from the Insituto de Hidrologia Meteorologia y Estudios Ambientales). The Colombian Departments of Valle de Cauca and Meta containing the cities of Cali and Villavicencio, respectively, experienced different Zika incidence between 2016–2017, with the Pan American Health Organization (PAHO) reporting upper estimates of 40 cases per 100,000 inhabitants in Meta and 605 cases per 100,000 in Valle de Cauca [23]. The study consisted of a series of 19 focus group discussions (FGDs) with female participants, and 14 one-to-one semi-structured interviews (SSI) with male participants. Participants fell into 3 categories: pregnant women, non-pregnant women of reproductive age, and their male partners. Pregnant and non-pregnant FGDs were held separately. In total, 10 FGDs were held with pregnant, and 9 with non-pregnant women. These FGDs took place in public institutions (such as libraries and health centers), contained between 2–11 participants (average of 7.5 per group) and lasted between 45–60 minutes. The focus group with only two participants was convened after the particular participants dropped out of their initially scheduled sessions. There were 14 one-to-one interviews with male participants, each lasting between 10–20 minutes. A total of 143 (71 pregnant and 72 non-pregnant) women, and 14 men consented to take part in the study. The female participants' ages ranged from 18 to 45 years, with an average age of 27.7 years. The male participants’ ages ranged from 19–58 with an average age of 36.7 years.

The study was designed to include participants from a range of lower and higher socio-economic strata [as defined by residential area, and designated according to the Colombian “estratos socioeconomicos” (socioeconomical strata) criteria] (**Table 1**). Recruitment was propositional, whereby organized groups containing potentially eligible participants were contacted by telephone, and all accepted agreed to attend. The groups contacted included: pregnant women’s groups, women's groups of sexual and reproductive health programs, and women's groups caring for children. For male SSI, recruitment priority was given to the partners of women participating in the FGDs. The inclusion criteria were as follows: pregnant women were required to be of legal age, were required to be pregnant (at any stage of gestational development), and needed to agree to participate in the study; non-pregnant women were required to be of legal and reproductive age, and needed to agree to participate in the study; males were required to be of legal age, and agreed to participate in the study. Written consent to take part in the study was obtained from all participants.

**Table 1:** Focus group composition. Income group uses a scale from 1–6, where 1 and 2 are low, 3 and 4 are middle and 5 and 6 are high—according to the Colombian “estratos socioeconomicos” (socioeconomical strata) criteria.

Focus group (n)	Location	Participant (n)	Pregnancy status	Income group(s)
1	Cali	10	Not pregnant	1
2	Cali	10	Not pregnant	1
3	Cali	8	Not pregnant	3
4	Cali	8	Not pregnant	1
5	Cali	8	Pregnant	1
6	Cali	7	Pregnant	1
7	Cali	8	Not pregnant	1,2
8	Cali	8	Not pregnant	4,5
9	Cali	7	Pregnant	1
10	Cali	8	Pregnant	3
11	Cali	9	Pregnant	3
12	Cali	2	Pregnant	4
13	Villavicencio	8	Not pregnant	2,3,4,5
14	Villavicencio	5	Not pregnant	4,5
15	Villavicencio	11	Pregnant	1,2,3
16	Villavicencio	8	Pregnant	1,2,3
17	Villavicencio	5	Pregnant	1,2,3
18	Villavicencio	7	Pregnant	1
19	Villavicencio	6	Not pregnant	3,4

### Interview structure

The interviewer in Cali was Carolina Mendoza, social worker and MSc student; and the interviewer in Villavicencio was Gloria-Isabel Jaramillo, PhD. Both interviewers were female and research scientists with a background in arboviruses at their respective universities. Both interviewers have had previous experience in performing FGDs for biomedical research. There was no relationship between the interviewers and the participants prior to commencement of the study. In Cali no people other than the interviewer, a field assistant and participants were present. In Villavicencio no people other than the interviewer, three observing medical students and the participants were present. Participants were introduced to the study and were told that the aim was to identify their concerns about Zika and their opinions around different personal protection methods. The FGDs were semi-structured and led by the moderator according to a predefined interview guide (see S1 Appendix). Interviewees were asked to speak freely, although prompts were provided if participants were reluctant to speak. They were conducted in Spanish, with the moderator asking the questions and allowing participants to answer directly and interact with the other participants. FGDs were either video recorded (Villavicencio) or tape recorded (Cali). Field notes were not taken. At the end of each session, recording was stopped and participants had the opportunity to ask the moderator questions if they had any concerns about Zika. They were also given a Zika fact sheet developed by the U.S. CDC.

### Data analysis

Focus group transcripts were analysed in their original Spanish by researchers at Universidad del Valle and Universidad de Cooperativa del Colombia, sede Villavicencio. English translations were produced, and were provided to researchers at the London School of Hygiene and Tropical Medicine (LSHTM) for independent analysis. Transcripts were analysed by thematic coding according to a pre-defined coding scheme. Coding was performed using the Atlas.ti (version 8.0) (Scientific Software Development Gmbh, Berlin) and NVivo (version 11.0) (QSR International, Melbourne) software packages. There were 7 coding families in total, representing: common mosquito control methods, arbovirus concerns, Zika knowledge, sources of Zika knowledge, changes to control behaviours during the Zika outbreak, ideal mosquito control tools, and views around PPT. Each theme family was divided into a larger number of sub-families. Each interview was independently analysed by at least three individual researchers, and results were compared and discrepancies discussed until a consensus was reached. As the purpose of using the two study sites was to increase diversity and not to directly compare the two cities, comparisons have only been drawn when clear differences between study sites emerged.

### Ethics

The study was approved by the ethical review boards of LSHTM (application number: 13592) and Universidad del Valle (Univalle: 004–018).

### Study limitations

Whilst conducting a partial retrospective study was necessary in order to gain insights into the knowledge and perceptions of Zika virus before and after the outbreak occurred in Colombia, studies of this kind can be problematic due to the reliance on participant memory and response. Responder opinions of their actions and awareness may be tainted by experiences that have occurred since the period they have been asked to reflect upon or the pressures felt at the time of being asked specific questions. These factors are likely to introduce responder and/or recall bias. The validity of this study is also at risk of ‘social interaction threat’, which is expected to occur within a focus group setting due to the social relationships and interactions present within this environment [24]. To negate issues associated with confirmability, the same data were analysed by multiple researchers. Despite this, due to the level of interpretation required to analyse qualitative data, there is still likely to be an element of researcher bias introduced by personal idiosyncrasies. In addition, issues around transferability may be apparent, since findings concluded are specific to the participants and may not be generalisable to alternate group in Colombia. Field notes were not taken and hence non-verbal information could not be considered. We believe this should not affect the results as recordings were available and transcripts were analysed by teams including the individuals performing the field work.

## Results

### The importance of Zika and other arboviruses

The magnitude of the perceived threat from the major arboviruses (dengue, Zika, chikungunya and yellow fever) varied considerably among study participants. Although the majority acknowledged the potentially serious consequences of arboviral infection, it was frequently described as less concerning than other infections, such as flu and diarrheal disease. However, some participants viewed arboviruses as a significant daily worry and this was often paired with a personal history of infection, or infection of a close family member or acquaintance.

“*No*, *they’re* [arboviruses] *alarming*. *When my son had dengue*, *his platelet count decreased in less than two hours”*. *(FGD3*, *Non-pregnant*, *Cali)*

Within the non-pregnant cohort, dengue, specifically severe dengue, was generally considered to be the arbovirus of greatest concern. Reasons given included a belief that dengue had the most serious acute symptoms, with a high probability of hospitalization, and the risks of mortality associated with severe dengue. Several respondents perceived a greater threat from dengue due to a belief that transmission was historically more consistent year-on-year, compared with the relatively emergent Zika and chikungunya epidemics, and, therefore, carried a greater risk of recurrence and infection. Similar to non-pregnant females, male participants were less likely to consider Zika as a primary arboviral concern, expressing greater fears over dengue and chikungunya.

“*I had Zika but it wasn’t so serious*. *I had joint pain but it wasn’t so severe”*. *(FGD4*, *Non-pregnant*, *Cali)*.

“*My feeling is that dengue is the most dangerous because it can be life-threatening*.*” (FGD13*, *Non-pregnant*, *Villavicencio)*.

“*At home we’re more concerned about chikungunya*, *classic dengue*, *and hemorrhagic*

Dengue [than Zika].” (Male interview 1, Villavicencio).

Pregnant women tended to be more likely than non-pregnant women to consider Zika the arbovirus of primary concern. Discussion among pregnant women often centred on concerns of the complications that can arise from congenital Zika transmission, particularly microcephaly and cognitive impairment.

*I think we all worry about viral diseases but we worry even more about Zika because…it affects primarily the foetus. (FGD11, Pregnant, Cali)*.

Chikungunya and yellow fever were least reported as the arbovirus of greatest concern in both the pregnant and non-pregnant groups. However, fears were frequently raised over the joint pain and arthralgia associated with chikungunya infection, with a belief that it carried the most severe chronic symptoms.

### Knowledge concerning Zika transmission and pathology

The majority of respondents in both the pregnant and non-pregnant groups reported an awareness of Zika as a disease threat and described mosquitoes as the source of transmission. However, the depth of knowledge beyond this varied considerably among participants, with some providing a description of Zika-specific symptoms and disease manifestations (e.g. birth defects), while others were able to give only a general arbovirus description (e.g. causes a rash, fever), or confused Zika with chikungunya, reporting a belief that they were alternate names for the same disease.

“*I thought chikungunya and Zika were the same*. *I’ve just realised that they’re different” (FGD4*, *Non-pregnant*, *Cali)*.

“*Very little [personal knowledge of Zika]* …*I can’t clearly make the difference between all of them [arboviral diseases] (Male interview 6*, *Villavicencio)*.

Women in the pregnant cohort were more likely than non-pregnant women to discuss congenital Zika, although awareness was not universal, with several participants, including some pregnant women claiming that the FGD was the first time they had be told about Zika-related congenital abnormalities.

“*I didn’t* [know about congenital Zika syndrome], *I’m so scared now that I’m going to carry a mosquito net everywhere” (FGD6*, *Pregnant*, *Cali)*.

*Pregnant woman (P1): “When I was hospitalized, a doctor told me that Zika is more dangerous in pregnant women because it mostly affects the unborn. The baby might be born with microcephaly: with deformed head and eyes*.”

*(P2): “Is it true? That’s scary*.”

*(Moderator): “What about the others?*”

*(P2): “I didn’t know*.”

*(P3): “We weren’t aware of all that*.”

*(Moderator)*: *“What about you*?*”* [to P4]

*(P4): “Actually, I didn’t know that either”. (FGD18, Pregnant, Villavicencio)*.

*Moderator: “What about Zika?*”

*Interviewee: “I don’t know what that is.*”

*Moderator: “Before I mentioned Zika, you have never heard of it?*”

*Interviewee: “No, I hadn’t” (Male interview 1, Cali)*.

Knowledge levels among the male partners were similarly variable, with some men showing familiarity with Zika-specific symptoms and transmission, while others claimed no knowledge beyond a basic awareness of it as an emerging disease.

Participants often acknowledged possessing a limited depth of knowledge of Zika. Several reasons were given for this, including a feeling of personal detachment from the disease:

*“Sometimes you see flyers at Health Promotions Agencies. However, as you haven’t been through that disease, you don’t read them”*, *(FGD1, Non-pregnant, Cali)*

and, a perception of insufficient community messaging on the part of the local health authorities.

“*I think the information* [distributed by local authorities] *was good enough but too fleeting*. *During the disease peak*, *they did give information*. *It was basic*, *though”*. *(FGD13*, *Non-pregnant*, *Villavicencio)*.

Few details were offered on the behavioural and visual characteristics of the vector, although it was frequently described as being ‘white-legged’, likely a reference to the white banding on the legs of *Ae*. *aegypti* and *Ae*. *albopictus*. Only one respondent correctly described the Zika-transmitting mosquito as being a day-biter. Few participants mentioned sexual contact as a possible transmission route.

Participants frequently reported television news broadcasts, private conversations with friends and family, and local health center messaging as the principal sources of information about Zika. A smaller number of participants described using the internet and social media as an information source, although it was noted by several respondents that the internet was often misleading and was generally considered to be unreliable. Information originating in local health centers, largely in the form of discussions with health workers and distributed leaflets, was thought to be particularly valuable, carrying greater authority than other messaging.

Numerous respondents reported receiving information from health authorities concerning mosquito bite prevention, with several describing attending seminars at health centres or schools, where messaging was reported to focus on larval management and the use of bednets. A minority of women, largely in the pregnant cohort, reported receiving information concerning sexual transmission, and described condom use as an appropriate precaution.

### Household mosquito abatement activities

Study participants were asked to discuss the mosquito reduction and bite prevention measures that they routinely implemented within their households. Measures were consistent between pregnant and non-pregnant cohorts, with most respondents reporting performing larval source management in and around their homes, including the regular emptying and cleaning of water containers to avoid stagnation, the covering of exposed water tanks, and the maintenance of a high level of general cleanliness, including the clearing from outside spaces of household refuse that could act as rainwater receptacles.

“*We really avoid water stagnation where mosquitoes might breed*. *We also avoid keeping containers with water*. *For example*, *after watering plants*, *many people leave the pot dishes full of water*. *We try to avoid all that”*. *(FGD1*, *Non-pregnant*, *Cali)*.

The idea of cleanliness as being key to avoiding mosquito proliferation was widely held, with a majority of participants considering the prevention of household clutter as an effective method for reducing mosquito densities in houses. Women rarely reported routinely applying topical mosquito repellents to themselves, although regular use on young children was more common. A reluctance to use repellents was often accompanied by comments describing repellents as being excessively expensive, at least for routine use, and this was particularly common in the groups containing participants from lower socio-economic strata. Women from lower socio-economic groups, particularly in Cali, were also more likely to report the topical use of products not manufactured for the purpose of repelling mosquitoes. Most notably, Vicks VapoRub, a cream sold as a nasal decongestant, was mentioned frequently by respondents as being routinely used as both a method of repelling mosquitoes and for the soothing of bites. Johnson’s Baby Oil and various other moisturizing body creams were also reported being applied as a protective barrier to mosquito biting.

*(P1) “I use Vicks VapoRub*. *I apply* [it] *on my skin*. *As it contains menthol*, *mosquitoes don’t approach”*.

*(P2) “I use it too, especially on my children. (FGD7, Non-pregnant, Cali)*.

Citronella oil was mentioned repeatedly throughout the FGDs, with participants reporting positive opinions of its efficacy and safety, with it being variously used as both a topical repellent and as a component of floor cleaner.

“*There’s also citronella repellent*. *It has no poison and is very good*.*” (FGD15*, *Pregnant*, *Villavicencio)*

Respondents reported burning various items of household refuse and dried plant material for the purpose of driving away mosquitoes from their homes. This practice appeared to be far more common in Cali, with participants describing the burning of egg cartons or eucalyptus seeds and leaves near windows on the street outside their homes. A smaller number of respondents reported burning specific insecticide-containing incense sticks and mosquito-coils.

“*I burn eucalyptus leaves in the backyard*. *A week ago*, *I did it and it repelled all the mosquitoes”*. *(FGD2*, *Non-pregnant*, *Cali)*.

Participants also described the routine spraying of the inside of their houses with insecticidal aerosol products, particularly Raid spray, and the use of plug-in insecticide disseminators. Other mosquito control methods frequently mentioned included the use of bednets and the use of a fan during the night.

Participants were asked to describe any alterations to their regular mosquito reduction and bite prevention activities in response to the Zika outbreak. Several respondents in both the pregnant and non-pregnant groups reported being so alarmed by Zika that they incorporated more rigorous mosquito abatement activities into their routines. Most commonly this involved intensified larval source management, but also included increased use of bednets and the more frequent use of insecticidal sprays.

“*Now* [since the Zika outbreak], *we sleep under a mosquito net*, *fumigate more often*, *and get rid of possible breeding sites like containers or bottles”*. *(FGD15*, *Pregnant*, *Villavicencio)*.

However, the majority of respondents reported making few changes to their regular mosquito abatement activities. This was often reported as being because the mosquito proliferation and bite prevention advice for Zika was the same as that disseminated in recent chikungunya and dengue outbreaks, and so people were familiar with the guidance and were, therefore, unmotivated to change their existing behaviours.

“*I personally haven’t changed anything*. *I come from Bogotá so I have always feared mosquitoes*. *We’re used to fumigating the house and*, *when chikungunya was on the rise*, *we even took vitamin B to avoid mosquito biting*. *In any case*, *we didn’t change our habits due to Zika”*. *(FGD13*, *Non-pregnant*, *Villavicencio)*.

### Protective clothing

Participants were asked to share their opinions on the personal use of clothing impregnated with mosquito repellents. Almost all were unaware that such products existed, although there was a strong sentiment from the majority of respondents that effective repellent clothing would be desirable and that they would wear such clothing, so long as it was safe, comfortable, and did not produce an unpleasant odor.

“*I’d wear it* [repellent clothing] *unless it’s warm*, *has a strong smell of chemical*, *or is sticky*. *I’d like something I’d be comfortable with”*. *(FGD7*, *Non-pregnant*, *Cali)*.

“*I would worry if I didn’t know the substance clothing was treated with*. *It could*, *for example*, *contaminate your blood or cause allergies*. *All skins are different*.*” (Male interview 5*, *Cali)*.

Few participants expressed a very strong dislike of the general concept of repellent clothing, with the majority of disapproving opinions resulting from a disinclination for additional contact with chemicals.

“*Although we use chemical products in our daily life*, *I insist on the idea that the fewer chemicals we use*, *the better*. *We should try to find other alternatives”*. *(FGD7*, *Non-pregnant*, *Cali)*.

A distinction was often made between different classes of repellent chemical, namely natural chemicals (usually plant-derived), and synthetic chemicals (those arising from chemical engineering processes). Synthetic repellents were generally considered to be less safe than those perceived as derived from plants. Citronella was often discussed as an example of how naturally-derived repellents could be a benign alternative to synthetic compounds. Several respondents considered the source of the active compound (whether it was regarded as naturally-derived or not) as more important than the overall repellent efficacy. That is, a perceived natural product with lower efficacy was considered more desirable than a synthetic product with higher efficacy.

“*I personally don’t like repellents*. *Rather than that*, *I prefer homemade natural products*. *They might not be as effective but*, *from my perspective*, *they’re less harmful*.*” (FGD13*, *Non-pregnant*, *Villavicencio)*.

Moreover, the smell of the repellent was a key concern. The presence of a perceived synthetic odour was considered by many women to be a major factor in determining whether a repellent product would be usable, with smells regarded as ‘natural’ considered far more acceptable.

“*I would* [wear repellent clothing] *if it smelled like citronella*, *not like something weird”*. *(FGD7*, *Non-pregnant*, *Cali)*.

During a Zika outbreak, pregnant women would be the primary target group for a repellent clothing product. Several women in the pregnant FGDs expressed anxieties over potential allergenic effects of a repellent chemical, or impacts on fetal development, with an element of scepticism around supplier claims relating to product toxicity. Several pregnant women reported believing that the most trustworthy distributor would be the local public health authority, and there was the sentiment that health center distribution would foster trust and improve uptake and usage rates.

Repellent clothing is most effective when the arms and the legs of the wearer are fully covered. Participants were asked to discuss the likelihood of their wearing full-length clothing if it had the potential to provide high levels of protection from arboviral diseases. Respondents tended to be strongly disinclined to wear clothes that fully covered arms and legs. While the potential benefits of wearing long-sleeved repellent clothing were generally accepted, the majority of participants considered long-clothing to be prohibitively impractical in the day-time heat.

“*In this heat*, *even if it’s true* [that protection was provided], *I’ll never wear long-sleeved clothes at home”*. *(FGD16*, *Pregnant*, *Villavicencio)*.

However, while some pregnant women explained that the heightened heat sensitivity during pregnancy would make long clothing too uncomfortable to bear, others suggested that the added protection would be the overriding factor and would make the discomfort of long-clothing worthwhile.

“*When you’re pregnant or have babies*, *if something is good for your baby’s benefit*, *you keep it even though it’s uncomfortable for you*. *So*, *at home*, *I don’t take off clothes that protect me from mosquitoes*.*” (FGD5*, *Pregnant*, *Cali)*.

The financial implications associated with purchasing additional products for protection against vector-borne diseases was a concern amongst study participants, particularly as costs were expected to be recurrent. Pregnant participants of low socioeconomic status were less inclined to wear repellent clothing everyday due to the additional expense that this would incur.

“*In a country like this*, *characterized by inequality*, *repellent should be included in the Mandatory Health Plan (POS)* [Plan Obligatorio de Salud], *at least during epidemic peaks so that it will reach the general public*. *I know repellent has a prohibitive price here”*. *(FGD14*, *Non-pregnant*, *Villavicencio)*.

Discussion around repellents by the female participants was occasionally framed in the role of a mother as having responsibility for the protection of her children. Some participants described being unable to afford repellents for themselves or their partners, but were willing to allocate money for repellents for their children.

## Discussion

The major burden of Zika morbidity is shared disproportionately by the children and families affected by congenital Zika syndrome. The targeting of pregnant women with enhanced bite-prevention measures in future outbreaks may provide additional protection to this high-risk group. The wearing of clothing impregnated with mosquito repelling compounds (such as the insecticide permethrin) could present an effective strategy [25], either on its own or used synergistically with topical repellents, and has been recommended by the U.S. CDC for protection from Zika infection [20]. Repellent clothing has also been proposed as a method of protecting children in areas endemic for dengue [26].

A number of laboratory studies have investigated the potential for using mosquito repelling compounds to protect against bites from *Aedes* mosquitoes. These include studies showing long-lasting protection from topical repellent applications [27, 28] and studies showing efficient protection from permethrin-impregnated fabrics [29], including promising results from experimental hut trials [30]. However, epidemiological data demonstrating impacts of impregnated clothing are still lacking. Mathematical modelling, considering populations of school children in Thailand wearing permethrin impregnated school uniforms, suggests that repellent clothing could have a significant impact on dengue transmission in that setting [31]. However, the model emphasized the importance of achieving high rates of uptake. There are a number of factors that could influence the uptake of a repellent clothing intervention, including an absence of disease awareness, a lack of full appreciation of disease risk, or anxieties over the safety of the repellent active ingredient itself [32]. Although concern among FGD participants was generally high for dengue, it was far more variable for Zika. The disparity between the diseases may be due, in part, to a greater historical familiarity with dengue. While Colombia has been experiencing epidemics of dengue for many decades, with thousands of cases of hemorrhagic dengue reported since the early 1990s [33, 34], Zika is relatively emergent, with the first cases reported in 2015. A perception of Zika as being of lower severity than dengue was also been reported among women of reproductive age in Peru, where Zika was ranked as the least severe of the mosquito-borne diseases [35]. Although pregnant women were generally better informed about Zika than non-pregnant women and were more likely to consider Zika to be the arbovirus of primary personal concern, the FGDs revealed that some pregnant women were completely unaware of the possibility of congenital Zika. This lack of basic knowledge about Zika was further highlighted by a frequent conflation by participants of the Zika and chikungunya viruses. A lack of knowledge about congenital effects of Zika virus was also found among residents of communities in Honduras, where only 40% of respondents reported an awareness of a causal relationship between Zika infection in pregnancy and microcephaly in newborns [36]. Moreover, although most participants were able to correctly identify the bite of a mosquito as the primary infection route, sexual transmission was seldom mentioned. Particularly low levels of knowledge of sexual transmission have been noted in several other studies examining respondents from various countries including the US [37], Brazil [38], Honduras [36] and Peru [35]. These results reveal gaps in awareness of some of important aspects of Zika transmission and pathology in Colombia, and highlight the need to increase and refine public health messaging.

Respondents reported performing a variety of routine mosquito bite prevention activities. A large proportion of study participants used environmental measures to reduce mosquito numbers around their homes, most frequently larval source management such as the emptying of water containers, which has shown to be effective when used as part of a broader integrated *Aedes* management strategy [39]. The use of insecticide-containing (commonly pyrethroid) plug-in evaporators and sprays, was also frequently reported. Although such products may be useful for reducing mosquito numbers inside a home, their effects on arboviral disease transmission are unclear due to a lack of published efficacy data with epidemiological outcomes. The use of personal protective measures was less common. While the use of topical mosquito repellents was occasionally described, this frequently referred to repellents such as citronella oil-based products or home-remedies such as Vicks VapoRub and moisturizing creams and lotions. These products have not undergone the extensive testing required to meet efficacy standards recognised by WHO and CDC criteria, and are, therefore, not recommended for use in arbovirus prevention. By using these products, rather than active ingredients that have been proven to provide protective efficacy, people are likely to be at a greater risk. The use of citronella oil and home-remedy repellents were often described by participants as preferable to synthetic chemical-based repellents due to a perceived lower chemical toxicity. Indeed, a sentiment throughout many of the discussions, particularly among pregnant women, was that products derived from plants were inherently safer than those based on synthetic chemicals. An elevated caution of pregnant women for repellents has been noted previously. A knowledge, attitudes and practices survey of pregnant women in Greece revealed a high proportion of participants reporting not using mosquito repellents because of fears they may be dangerous for the foetus [22]. This strongly suggests that educational messaging does not sufficiently emphasize the low toxicity and high efficacy of many of the recommended chemically synthesized repellents, and it may be possible to improve uptake rates by increasing the distribution of safety information on these compounds. DEET, for example, is approved for use by pregnant women by the U.S. EPA, and its use has been recommended by the U.S. CDC as a tool for reducing Zika transmission [20]. Moreover, safety and toxicology reviews of DEET have concluded that it possesses low acute toxicity and is safe for pregnant women [40, 41]. Alternatively, there is one highly effective plant-based repellent recognised by the CDC [18], that may be more appealing to some pregnant users. PMD (p-menthane 3,8-diol) is a repellent purified from oil of lemon eucalyptus, and has been shown to have repellent properties similar to that of DEET [42]. Public awareness campaigns and health provider advice need to stress the importance of using repellents that contain active ingredients that have been evaluated for efficacy and approved by appropriate authorities, and emphasize the safety profiles of repellents recommended for use during pregnancy.

Overall, study participants were positive about a potential role for repellent clothing in protecting against Zika and other arboviral diseases, provided the active ingredients were considered safe and the clothing comfortable. Currently, permethrin, a pyrethroid insecticide, is the only repellent registered for the treatment of fabrics in the U.S. Although the safety data available for permethrin use during pregnancy is not as extensive as that for DEET, the World Health Organization (WHO) considers topical application of permethrin for scabies treatment compatible with breastfeeding [43]. However, there are a very limited number of animal studies suggesting that prenatal pyrethroid exposure may cause adverse effects on neural development, although the doses used in these experiments tend to be far higher than those expected during normal human exposure [20, 44]. The presumed low risk of this compared to the extreme fetal pathologies of Zika led the U.S. CDC to recommend permethrin impregnated clothing as a personal protective measure for pregnant women against infection [20, 45].

However, achieving meaningful uptake of repellents and pyrethroid-impregnated clothing is likely to be a challenge even in the presence of enhanced messaging and education. A variety of social factors have been correlated with improved vector borne disease protection knowledge and practices and include high educational status, living in a stand-alone house and having a more advanced knowledge of a disease [46]. Participants identified community health workers as being particularly trusted sources of information, suggesting that teams comprising trained community members could provide educational sessions to pregnant women in future outbreaks. Furthermore, the capacity to implement appropriate personal mosquito control interventions is also based on an individual’s economic capacity to purchase control products [47]. Some pregnant participants described not having sufficient income to purchase repellents, or reported allocating money to provide repellents for their children, while being unable to afford repellents for themselves or their partner. Similarly, a large proportion of women in a survey in Peru felt that the only preventative action for Zika was larval source reduction, since repellents were too expensive [35]. At risk populations may often be aware of the benefits of repellents, but may not necessarily have the financial means to purchase products and implement effective personal protection. To increase coverage, the federal government in Brazil implemented free distributions of insect repellents to low-income/at-risk individuals [48]. A crucial element in the optimisation of PPT uptake will, therefore, be a robust evaluation of the influences of socio-economic factors in determining access to educational resources and control products.

Despite government regulations to manage arbovirus transmission in Colombia, outlined as vector control actions (mainly indoor and outdoor fumigation) in National Institute of Health guidelines, the incidence of arboviral disease has continued to increase. This may be due, in part, to the top-down approach taken by the eradication programmes, with little consideration for community engagement or local health worker participation [49]. Overlooking local knowledge and socio-political dynamics is likely to result in reduced coverage and negatively affect intervention impact. Indeed, several discussion group participants indicated a desire to engage directly with local health workers on vector control issues, calling for more control and an active role in preventing transmission.

The wearing of long-sleeved and legged clothing was considered by the majority of participants to be impractical in the high day-time temperatures. This has been previously reported by individuals in tropical climates. A survey of women in Puerto-Rico, for example, found that the wearing of long-sleeved clothing for bite prevention during the Zika outbreak was very infrequent, with the most common reason given as it ‘being too hot’ [50]. A variety of different preferences for acceptable clothing materials and designs were discussed by FGD participants, indicating that the design and distribution of repellent garments with broad user appeal would be logistically challenging; a simpler and potentially more successful approach may involve the development of a system whereby repellency could be generated by a user on their own clothing, e.g. a textile spray or a laundry additive.

Ensuring high uptake of a bite prevention technology in the high-risk group relative to the general population may be necessary for effective implementation of a PPT strategy. If a PPT technology reached high uptake among the general population, there may be a risk of deflecting mosquito bites on to those less inclined to use or less able to obtain such products. If members of the high-risk group were less likely to use the PPT than the general population, the incidence of severe disease could actually increase. This is a potential concern in the case of Zika, where pregnant women tend to be warier of using repellent products than the population at large [22] and should be considered if PPT is to be recommended for use at scale.

Although several vaccines are currently in development, the repertoire of tools immediately available for controlling Zika transmission is extremely limited. PPT represents one of the few readily deployable interventions for reducing biting, although achieving meaningful rates of uptake remains a formidable challenge. A very limited number of studies have been carried-out directly assessing attitudes and practices concerning PPT use. Developing a more nuanced understanding of the concerns and misconceptions that contribute to low PPT uptake rates through engaging with target users is crucial for optimising messaging and improving the appeal of repellent products. The focus group discussions in the present study revealed a high-risk population in Colombia with variable knowledge of Zika and its transmission, with many participants reporting little to no awareness of the virus. Many of the study participants were also highly sceptical of repellent safety. Taken together, these data suggest that although achieving behaviour change is clearly highly complex, involving societal, economic and political factors, many of the issues influencing uptake stem from a lack of awareness of disease risk and misconceptions about repellent safety. In the case of Zika, both issues could be addressed through targeted information dissemination to high-risk individuals, for example, during antenatal sessions.

## Conclusions

Given the importance and challenges of achieving high uptake with topical repellents and repellent clothing among pregnant women, this study attempted to identify areas where product design and dissemination can be optimized to maximise target-user uptake and compliance. The FGDs revealed that not all pregnant participants were aware of the full range of pathologies associated with Zika, and worryingly, several did not know about the possibility for congenital transmission and fetal birth defects. While knowledge of mosquito transmission appeared universal, sexual transmission was mentioned rarely. Equitable access to repellents was often raised as a concern, particularly in the lower socioeconomic groups, with several participants indicating that, for them, regular use of commercially-available repellents would not be financially viable. Significant worries were raised that there may be health risks associated with repeated contact with repellent chemicals, particularly during pregnancy. The use of repellent products that do not contain recommended active ingredients also appears to be common, with the view that these products present lower toxicity. Participants viewed plant-derived repellents as possessing inherently less toxicity than synthetic repellents, and identified health workers as particularly trusted sources of information on bite-prevention.

The results of this study highlight significant gaps in the knowledge of key aspects of Zika disease and bite-prevention among women in Colombia. The discussions suggest that bite-prevention education campaigns need to emphasize the risks of congenital Zika, stress the positive safety profiles of repellents registered for use in pregnancy, and raise awareness of the benefits of using repellent technologies that have been efficacy-tested and approved by recognized regulatory authorities.

## Supporting information

S1 Appendix Interview guide used in the focus groups. (DOCX)
