# Supplementary material for: An investigation into the knowledge, perceptions and role of personal protective technologies in Zika prevention in Colombia
Source: PLoS Negl Trop Dis. 2020 Jan 21;14(1):e0007970. doi: 10.1371/journal.pntd.0007970 (PMC7010294; doi:10.1371/journal.pntd.0007970)
Supplement: S1 Appendix — (DOCX) [file pntd.0007970.s001.docx]

S1 Appendix

Interview guide.

1. Mosquito control by households and the community

a. What do you do in your household in order to reduce the number of mosquitos living in the area, and the number of bites that you and your family receive?

b. Is there any sort of community effort to reduce mosquito breeding sites?

2. Mosquito control by local authorities

a. What mosquito control activities, if any, are undertaken by the local authorities in your community?

3. Concern about mosquito-borne diseases

a. In relation to the various issues that you and your family have to manage in your daily lives, how much are you concerned about mosquito-borne diseases such as dengue, chikungunya, yellow fever, and Zika?

b. Are these four diseases of equal concern to you, or is one of more concern than the other three?

4. Knowledge about Zika

a. Turning specifically to Zika, do you know anyone personally who has had Zika?

b. If so, what is your relationship with them?

c. What do you know about Zika?

d. Are there any aspects of the disease that you would like to know more about?

5. Sources of knowledge about Zika

a. Where have you received your knowledge about Zika from? (probes to include social media)

b. Which of the information sources about Zika do you think have been the best, and which have been the least useful?

6. Zika messages

a. What are the main messages that you have received from the authorities about Zika?

b. Have these messages been helpful for you, or not?

7. Changes in mosquito control practices

a. Have there been changes in mosquito control practices in your community, and in your own personal protection, since the emergence of Zika?

b. What did you do before Zika?

c. What do you do now?

8. Preferred mosquito control activities

a. The Zika mosquitoes bite during the day. Given this, what types of mosquito control would you like to see?

9. Personal protection interventions

a. What are your thoughts on personal protection interventions, such as mosquito-repellent clothing?

b. How likely would you be to use these and what would be your considerations?
